# Impact of age and clinical factors on the feasibility of mobile digital monitoring in people at risk of suicide

**DOI:** 10.1371/journal.pone.0346772

**Published:** 2026-04-16

**Authors:** Ismael Conejero, Ana María de Granda-Beltrán, Lucía Albarracín-García, Alejandro Porras-Segovia, María Luisa Barrigón, Jorge Lopez-Castroman, Phillippe Courtet, Antonio Artés-Rodriguez, Enrique Baca-Garcia, Memind Group

**Affiliations:** 1 Department of Psychiatry, Nimes University Hospital, Nimes, France Institut de Génomique Fonctionnelle, University of Montpellier, CNRS-INSERM, Montpellier, France; 2 Instituto de Investigación Sanitaria Fundación Jiménez Díaz, Madrid, Spain; 3 Universidad Autónoma de Madrid, Madrid, Spain; 4 Department of Psychiatry, Hospital Universitario Fundación Jiménez Díaz, Madrid, Spain; 5 Departamento de Psiquiatría, Hospital Rey Juan Carlos Móstoles, Madrid, Spain; 6 Institute of Psychiatry and Mental Health, Hospital General Universitario Gregorio Marañón, IISGM, Madrid, Spain; 7 Department of Psychiatry, Radiology, Public Health, Nursing and Medicine; University of Santiago de Compostela, A Coruña, Spain; 8 Department of Emergency Psychiatry and Acute Care, Centre Hospitalier Universitaire Montpellier, University of Montpellier, Montpellier, France; 9 Department of Signal Theory and Communications, Carlos III University, Madrid, Spain; 10 Departamento de Psiquiatría, Universidad Autónoma de Madrid, Madrid, Spain; 11 Universidad Católica del Maule, Talca, Chile; 12 Departamento de Psiquiatría, Hospital Central de Villalba Villalba, Madrid, Spain; 13 Departamento de Psiquiatría, Hospital Universitario Infanta Elena Valdemoro, Madrid, Spain; 14 Centro de Investigación Biomédica en Red de Salud Mental, Madrid, Spain; 15 CIBERSAM, Research Group, Madrid, Spain; 16 Department of Psychiatry, Nimes University Hospital, Nimes, France; University of Catania, ITALY

## Abstract

**Objective:**

Assessing the risk of suicidal outcomes is challenging, particularly in older people. Smartphone-based digital phenotyping may help to monitor suicide risk through ecological momentary assessment (EMA) applications. In this real-world study, we investigated if age and other clinical factors were associated with participation in EMA at baseline, and with retention in EMA monitoring among patients at risk of suicide.

**Methods:**

Participation in EMA was determined by quantifying the installation of the MEmind mobile application in individuals involved in the SmartCrisis 1.0 and 2.0 studies. The patients were followed-up over a 6-month period.

**Results:**

N = 512 patients met inclusion criteria, of which 387 installed the MEmind application on their smartphone. While age as a continuous variable was not associated with using EMA at baseline, being aged older than 50 and being engaged in an intimate relationship were independently associated with longer participation in EMA (OR 2.070, 95%CI [1.054–4.066], and OR 2.103, 95%CI [1.076–4.110], respectively). In an exploratory survival analysis, we found that EMA retention increased with age (p < 0.001).

**Conclusion:**

Feasibility of EMA seems warranted in older people at risk of suicide. Clinicians should be encouraged to offer EMA monitoring to older adults, as they commonly face limitations in their access to healthcare facilities.

## Introduction

Suicide risk increases with age [1]. Among people aged between 50 and 69 years, the global suicide rate is 16 per 100 000, while it reaches 27 per 100 000 among those aged 70 and older [2]; [3]. Suicide attempts have mostly increased among young individuals in the USA in recent decades [4]. In contrast, deaths by suicide have increased among older people, rising by 6.6 per cent in the 45–64 age group, and 8.1 per cent in the 65 + age group between 2021 and 2022 [5]. In spite of these figures, many older suicide attempters do not receive appropriate psychiatric care after self-harm in the United States [6]. The situation is equally concerning in other countries, such as Spain, were the incidence of suicide attempts among older adults has been estimated at 35 per 100 000 [7].

Early detection of suicidal behavior and tailored monitoring of people at risk is essential to prevent death by suicide, especially in the ageing population. Particular attention should be paid to the post-discharge period, as the risk of suicide increases after hospitalization [8]. In addition, social isolation and the feeling of loneliness are well-established risk factors for the occurrence of suicidal ideation in older patients [2].

Nonetheless, predicting suicide attempts and deaths by suicide remains a challenge. Among older people, physical disability, cognitive impairment and poor access to psychiatric facilities may alter accurate and timely psychological assessment [9]. Individuals aged 50 and older may have more limited access to preventive measures, such as engagement in safety planning [10]. Therefore, the accurate recording of dynamic health data in daily life may improve the identification of suicide risk. Mobile health (mHealth) platforms help identify suicide risk in the short term [11], and are generally well accepted in the psychiatric population [12]. Smartphone-based digital phenotyping and mHealth provide an innovative framework for ecological momentary assessment (EMA) [13].

EMA has received increasing interest in suicide research [14]. EMA involves real-time monitoring of mental health in daily life (psychological state, mood), based on active patient participation. It integrates contextual stressors which are interpreted within an ecological framework.

However, the so-called digital divide may affect the participation in EMA [15], as the individuals born before 1970 have only recently become familiar with the internet and mobile technologies. In fact, people born prior 1960, and those belonging to the sixties generation were middle aged when world wide web was popularized in 1994 [16], and when the first smartphone was released in 2007 [17], respectively. In addition, repeated assessment and inadequate software design may reduce the acceptability of EMA protocols due to the burden on daily life. Fatigue or apathy associated with mental disorders and difficulties in using new mobile technologies may alter the retention rate in EMA programs over time. Previous studies exploring the feasibility of EMA have reported heterogeneous compliance rates, ranging from 36 to 98% in older adults [18]. The majority of feasibility studies were conducted with small sample sizes or did not evaluate patient retention beyond two weeks [12,18,19]. A recent systematic review reported median sample sizes of 53 participants [20]. Moreover, most studies are monocentric or conducted in one country. Finally, patients receiving financial incentives were often included, which may artificially increase retention rates [14].

Porras-Segovia and colleagues (2022) evaluated and compared the feasibility of active and passive EMA in suicide attempters enrolled in the SmartCrisis 1.0 study [21]. They found that younger age and being employed were associated with a higher acceptance to install at least one active or passive EMA application. They also reported that the retention rate for the use of active EMA fell below 50% after 3 months of follow-up, and that retention was determined by other independant factors including the exclusion of passive EMA and the satisfaction with the application. However, participation in active EMA was not evaluated specifically.

A better understanding of participation in active EMA according to age and other patient characteristics may help to design well-tailored EMA programs and improve their effectiveness for suicide prevention [18]. Improving the retention of older people in active EMA for suicide prevention is important, as they often experience difficulties to access standard psychiatric healthcare [22]. Multicentre real-world studies including large patient samples, followed-up over an extended period, without financial incentives and focusing specifically on active smartphone-based EMA are currently lacking.

This study examined whether age and other clinical characteristics were associated with participation in active EMA at baseline, defined as application installation, in a large sample of patients at risk of suicide. We then explored the association between those characteristics and retention rates over time in EMA participants. We hypothesize that age and other factors involved in the digital divide, such as educational level, will affect retention in EMA.

## Materials and methods

### Context and design

This is a feasibility study, based on data from the SmartCrisis 1.0 (NCT03720730), and the SmartCrisis 2.0 (NCT04775160) studies, conducted in Madrid, Spain and in Montpellier and Nîmes, France [23]; [24]. The SmartCrisis studies involve the use of an application “MEmind” designed to monitor mental health parameters. These applications were installed on the patient’s smartphone by a trained psychologist during the first visit. Patients were instructed on how to use them. The EMA application collects explicit data and relies on the active participation of the patients. Information collected include questions prompted in patients’s Smartphones on day to day mood and negative feelings, wish to die, sleep quality and quantity, and appetite. Patients were followed-up over 6 months. The protocols were approved by the Institutional Review Board of the Fundación Jiménez Díaz Hospital, (Madrid, Spain) and by the local ethics committee (Comité de protection des Personnes OUEST IV, Nantes, France, 20187-A02634-49). All participants provided written informed consent before entering the study. All experimental methods were carried out in accordance with the ethical guidelines determined by the Declaration of Helsinki. The recruitment started in April 2018 in Madrid, and in July 2018 in the French centres.

### Sample

The patients were recruited consecutively in the Emergency Department and Outpatient Mental Health Clinic of the University Hospital Fundación Jiménez Díaz (Madrid, Spain), in the Centre Hospitalier Universitaire (CHU) of Montpellier (France), and in the Centre Hospitalier Universitaire (CHU) of Nîmes (France). Participants received no financial incentive. Inclusion criteria were: age 18 years and older, a history of suicidal thoughts or behaviors assessed with the Columbia Suicide Severity Rating Scale (CSSRS) [25], owning a smartphone with an iOS or Android operating system with an internet connection, and being able to give written informed consent [23]; [24].

### Sociodemographic and clinical data collection

At baseline, sociodemographic characteristics were collected, including age, sex, marital status, employment status, and educational level. Clinical diagnosis was assessed from the electronic clinical records in accordance with the International Classification of Diseases, 10th Revision (ICD-10) criteria [26]. Suicidality was evaluated using the CSSRS and the suicidality module of the MINI International Neuropsychiatric Interview 7.0.0 [27]. Depression was investigated with the clinician version of the Inventory for Depressive Symptomatology (IDS-C) [28], and impulsivity was assessed through the Barrat Impulsiveness Scale [29].

### Outcomes

EMA feasibility was defined by installation of the MEmind application on the smartphone, taken as an indicator of participation. Installing the application is a key factor for patients’ access to EMA monitoring. Prior studies assessed the willingness to download a smartphone application to evaluate the acceptability of EMA [30], and recorded installation rates to investigate the feasibility of active EMA in psychiatric and student populations [12]. We then compared age and patients’ characteristics according to the installation or not of the application. Retention rate was assessed over the 6 month follow-up. Retention in EMA was considered acceptable if the application remained installed for at least 90 days following enrollment. This conclusion aligns with Porras-Segovia et al. (2022), who observed retention rates dropping below 50% after 3 months. Additionally, the 90-day threshold corresponds to the period with the highest suicide risk following a suicide attempt or post-discharge [31]; [32]. Furthermore, the 90-day retention period was investigated in prior feasibility studies of digital monitoring from our group [33].

We defined the > 50 age category based on the potential effects of the digital divide [15], on the late acculturation with smartphone [17], and on the limited access to effective suicide prevention in this population [10]. For exploratory analysis, we defined four age categories 18–35; 35–50; 50–60 and >60 as defined by the by the American Psychological Association [34] and by the World Health Organization [35].

### Statistical analysis

Clinical and sociodemographic characteristics of participants are presented as numbers and corresponding percentages for categorical variables and mean (Standard Deviation, SD) or median [25th percentile – 75th percentile], depending on the distribution for quantitative variables. Clinical and sociodemographic characteristics were compared between EMA participants and non-participants using χ^2^ or ANOVA. Significant variables were then entered into a logistic regression model. Survival curves in different age ranges were designed with Kaplan-Meier and log-rank tests. Statistical analysis was performed using SPSS 29.0 software. The significance level was set at p < 0.05.

## Results

### Baseline characteristics of the sample

**Fig 1:**
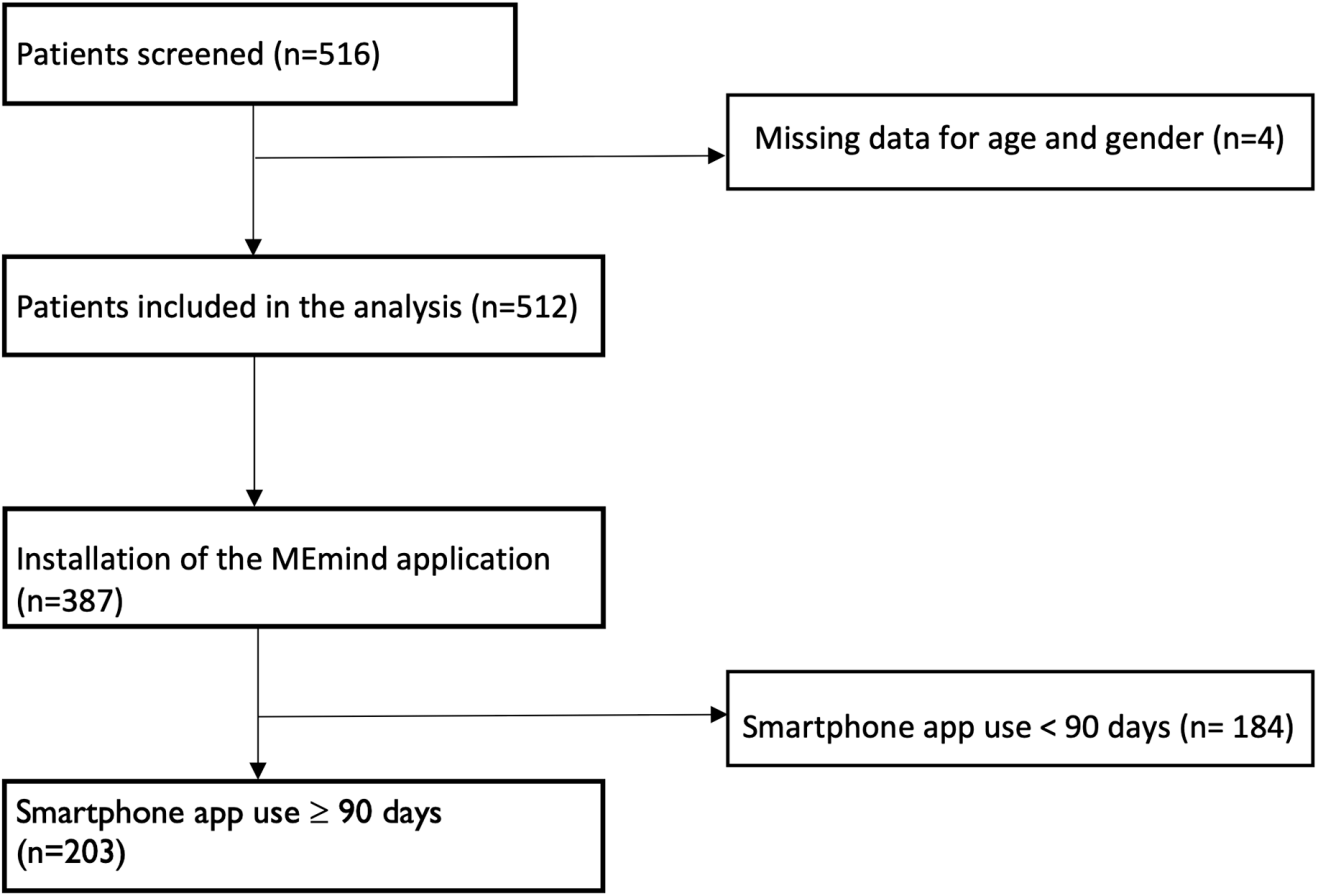
Flow chart of the study.

From the 516 patients evaluated at inclusion, 4 were excluded because of missing data regarding age and gender variables. Upon the 512 patients included in the analysis at baseline, 387 had installed the MEmind application on their smartphone. The flow chart is provided in Fig 1.

Clinical characteristics of the sample at baseline are shown in Table 1. The mean age was 40.9 years [SD = 15], and 182 of the patients were male (35.5%). Most patients had completed higher education (n = 302; 74.8%) and had been in an intimate relationship, either currently partnered (n = 159; 31.6%) or separated/widowed (n = 108; 21.5%). Most patients were unemployed (n = 233; 73%) and had a major depressive episode according to the IDS-C (n = 422; 82.4%). In our sample, 150 patients had attempted suicide in their lifetime (43.2% of the 347 patients with available data).

**Table 1:** Clinical characteristics of patients at baseline.^a^

Clinical characteristics	Whole population (n = 512)	Missing data	App installed (n = 387)	App not installed (n = 125)	Significance (p-value) ^b^
**Age (years)**	40.9 [15]	–	40 [14]	43 [17]	0.11
**Age (>50 years)**	159 (31.1%)	–	114 (22.3%)	45 (8.8%)	0.17
**Gender (male)**	182 (35.5%)	–	129 (25.2%)	53 (10.4%)	0.07
**Study level**		108			0.003
No/ Primary/ Secondary studies	102 (25.2%)		68 (16.8%)	34 (8.4%)	
Higher educational degree	302 (74.8%)		245 (60.6%)	57 (14.1%)	
**Family status**		9			0.17
Single	236 (46.9%)		175 (34.8%)	61 (12.1%)	
Separated or widowed	108 (21.5%)		78 (15.5%)	30 (6%)	
Lives with a partner	159 (31.6%)		129 (25.6%)	30 (6%)	
**Employement**		193			0.01
Employed	57 (17.9%)		36 (11.3%)	21 (6.6%)	
Unemployed	233 (73%)		179 (56.1%)	54 (16.9%)	
Retired	29 (9.1%)		16 (5%)	13 (4.1%)	
**Major depressive disorder**		–			<0.001
(IDS-C < 13)^c^	90 (17.6%)		50 (9.8%)	40 (7.8%)	
(IDS-C > 13)	422 (82.4%)		337 (65.8%)	85 (16.6%)	
**Lifetime suicidal behavior**		165			0.59
No	197 (56.8%)		152 (43.8%)	45 (13%)	
Yes	150 (43.2%)		112 (32.3%)	38 (11%)	
**Suicidal behavior in the last month**					
No	199 (70.3%)	229	150 (53%)	49 (17.3%)	0.51
Yes	84 (29.7%)		64 (22.6%)	20 (7.1%)	
**Impulsivity level (BIS, Median score = 69)** ^**c**^					
Low (<median)	120 (51.7%)	280	106 (45.7%)	14 (6%)	0.58
High (> median)	112 (48.3%)		99 (42.7%)	13 (5.6%)	

^a^ Data are means [Standard Deviation], or number (%).

^b,^ The characteristics of patients with the MEmind app installed on their smartphone were compared with those without the installed app.

^c.^ IDS-C: Inventory for Depressive Symptomatology; BIS: Barrat impulsiveness scale

### Association between age and EMA participation at baseline

At baseline, age did not significantly differ between EMA participants (mean age = 40 [SD = 14]) and non-participants (mean age = 43 [SD17]), p = 0.11.

### Association between other patients’ characteristics and EMA participation at baseline

Univariate analysis shows that EMA participants were more likely to have a higher level of education (60.6% vs 14.1%; p = 0.003), more likely to be unemployed (56.1% vs 16.9%, p = 0.01) and more likely to be depressed (65.8% vs 16.6%; p < 0.001) than non-participants (Table 1). No relationships was found with the gender, with lifetime or last-month suicidal behaviors, nor with the level of impulsivity. Moreover, there were no associations between EMA participation and psychiatric comorbidities, including any substance use disorder, bipolar disorders, or general anxiety disorders, nor with the participants’ country of origin (i.e., Spain or France) (Data not shown). Significant univariate associations were tested in two separate logistic regression models, including either educational level (model 1) or employment status (model 2), as those variables are highly colinear (p < 0.001). The results of the logistic regression analysis are shown in Table 2. In model 1, higher educational level and major depressive disorder were associated with more frequent EMA participation (OR = 2.289, 95%CI [1.362–3.846], and OR = 3.017, 95%CI [1.640–5.551], respectively). In model 2, unemployment and major depressive disorder were associated with more frequent EMA participation (OR = 2.226, 95%CI [1.168–4.246], and OR = 2.798, 95%CI [1.468–5.333], respectively).

**Table 2:** Clinical characteristics associated with EMA app installation at baseline.

	Model 1			Model 2		
**Clinical characteristics**	**Estimated odds ratio (Exp(b))** ^**a**^	**95% CI for Exp(b)**	**Significance** **(p-value)**	**Estimated odds ratio (exp(b))**	**95% CI for Exp(b)**	**Significance** **(p-value)**
**Age > 50 (years)**	1.024	0.603 - 1.740	0.930	0.630	0.354 - 1.122	0.117
**Gender (male)**	0.616	0.376 - 1.007	0.054	0.870	0.520 - 1.458	0.598
**Higher educational degree** ^**b**^	2.289	1.362 - 3.846	0.002	–	–	–
**Employment (Model 2)**						
Employed	–		–	–		
Unemployed	–		–	2.226	1.168 - 4.246	0.015
Retired	–		–	1.376	0.477 - 3.973	0.555
**Major depressive disorder (IDS-C ≥ 13)** ^**c**^	3.017	1.640 - 5.551	<0.001	2.798	1.468 - 5.333	0.002

a. The association between the characteristics of patients and the installation of the MEmind app was tested in a logistic regression model.

b. The study level was significantly associated with employment status p < 0.001, hence these variables were tested in separate models (Study level in model 1 and employment status in model 2).

c. IDS-C: Inventory for Depressive Symptomatology

### Association between age, patients’ characteristics and EMA retention over time

We then assessed the association between patients’ characteristics at baseline, and EMA retention over the follow-up. Univariate analysis shows that patients participating in EMA for more than 90 days were more likely to be aged 50 years or older (20.2% vs 9.3%, p < 0.001), to be in an intimate relationship (20.2% vs 13.6%, p < 0.001), and to be unemployed (44.6% vs 32.9%, p = 0.023) than those participating for less than 90 days (Table 3).

**Table 3:** Clinical characteristics according to smartphone app use < 90 days or ≥90 days.^a^

Clinical characteristics	Missing data	Smartphone app use < 90 days (n = 184)	Smartphone app use ≥ 90 days (n = 203)	Significance (p-value)^b^
**Age**	–			< 0.001
(<50 years)		148 (38.2%)	125 (32.3%)	
(≥50 years)		36 (9.3%)	78 (20.2%)	
**Gender**	–			0.472
Male		58 (15%)	71 (18.3%)	
Female		126 (32.6%)	132 (34.1%)	
**Study level**	74			0.154
No/ Primary/ Secondary studies		38 (12.1%)	30 (9.6%)	
Higher education		113 (36.1%)	132 (42.2%)	
**Family status**	5			<0.001
Single		103 (27%)	72 (18.8%)	
Separated or widowed		27 (7.1%)	51 (13.4%)	
Lives with a partner		52 (13.6%)	77 (20.2%)	
**Employement**	156			0.023
Employed		24 (10.4%)	12 (5.2%)	
Unemployed		76 (32.9%)	103 (44.6%)	
Retired		6 (2.6%)	10 (4.3%)	
**Major depressive disorder**	–			0.945
(IDS-C < 13)^c^		24 (6.2%)	26 (6.7%)	
(IDS-C > 13)		160 (41.3%)	177 (45.7%)	
**Lifetime suicidal behavior**	123			0.459
No		69 (26.1%)	83 (31.4%)	
Yes		56 (21.2%)	56 (21.2%)	

^a^ Data are numbers (%).

^b,^ The characteristics of patients with the MEmind app installed were compared between those who used the app < 90 days and ≥90 days.

^c.^ IDS-C: Inventory for Depressive Symptomatology

Statistically significant univariate associations were then analyzed in a logistic regression model (Table 4). Being aged 50 years or older and currently living with a partner were independently associated with longer EMA participation (OR = 2.070, 95%CI [1.054–4.066], and OR = 2.103, 95%CI [1.076–4.110], respectively).

**Table 4:** Clinical characteristics of the patients associated with EMA app use ≥ 90 days during follow-up.

Clinical characteristics	Estimated odds ratio (exp(b))^a^	95% CI for Exp(b)	Significance (p-value)
**Age (≥ 50 years)**	2.070	1.054 - 4.066	0.035
**Gender (male)**	1.128	0.642 - 1.982	0.675
**Family status**			
Single	–	–	–
Separated or widowed	1.830	0.839 - 3.990	0.129
Lives with a partner	2.103	1.076 - 4.110	0.030
**Employment**			
Employed	–	–	–
Unemployed	2.031	0.908 - 4.544	0.085
Retired	1.180	0.305 - 4.562	0.810

a, The association between the characteristics of patients and the use of the MEmind app during follow-up was tested in a logistic regression model.

### Exploration of EMA retention over time according to age: survival analyses

**Fig 2:**
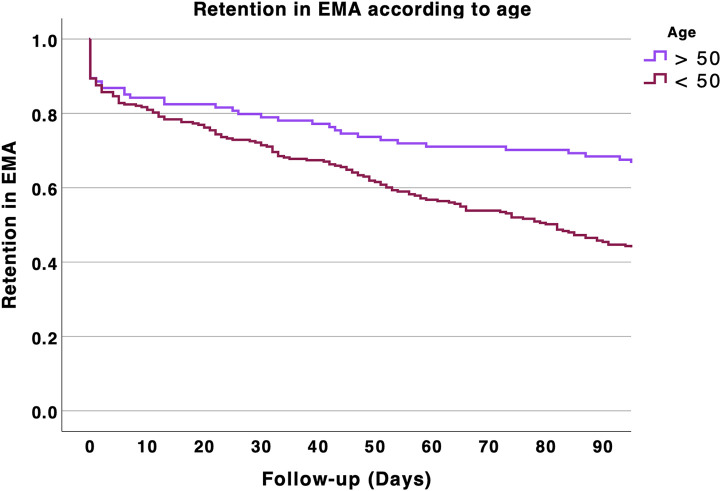
Retention rates in EMA over the 3 months of follow-up according to age. Age was divided in two categories: 18-50 years and > 50 years old.

**Fig 3:**
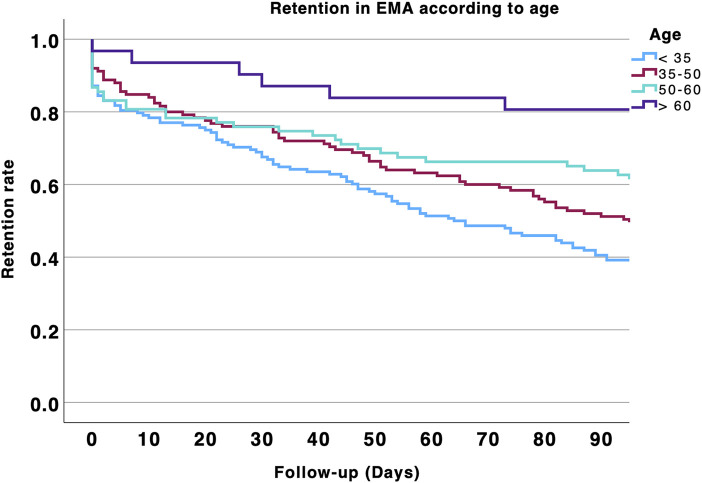
Retention rates in EMA over the 3 months of follow-up according to age. Age was divided in four categories: 18-35; 35-50; 50-60 and > 60 years old.

**Fig 4:**
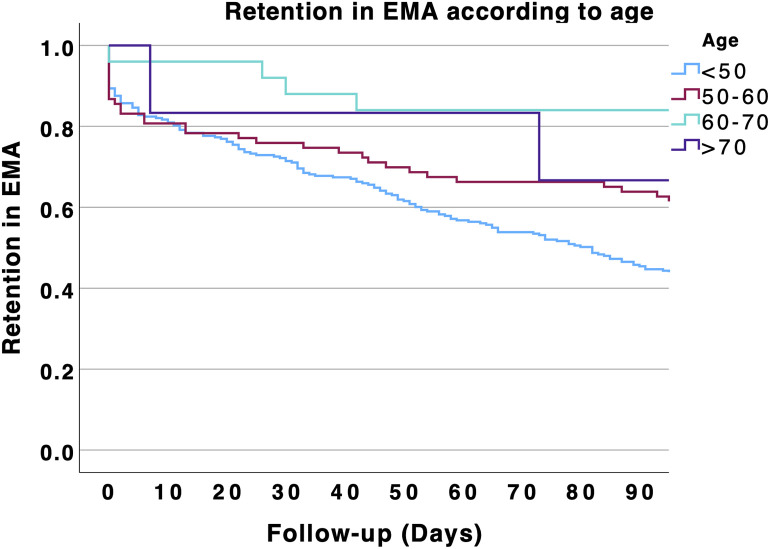
Retention rates in EMA over the 3 months of follow-up according to age. Age was divided in four categories: < 50; 50-60; 60-70 and > 70 years old.

In an exploratory survival analysis, we found that patients aged 50 years and older were more likely to participate in EMA > 90 days compared with those younger than 50 (p < 0.001). The results are shown in Fig 2. We also explored EMA retention according to young (18–35 years), middle (35–50), or older age ranges (50–60 and > 60 years, Fig 3). Survival analysis showed that EMA retention increased with age (p < 0.001). However, EMA retention decreased in individuals aged 70 and older (p < 0.001, Fig 4). Specifically, participation in EMA for more than 90 days increased with age: 45.8% in patients under 50 years, 63.9% in those aged 50–60 years, 84% in those aged 60–70 years, and 66.7% in those aged 70 years and older.

## Discussion

In this study, we explored the impact of age and other patient characteristics on EMA participation and retention in a large sample of patients with a history of suicidal thoughts or behaviors. At baseline, EMA participation was not associated with age but was more frequent among patients with higher education, unemployment, and major depressive disorder. During the follow-up, older age and being in couple predicted EMA use over a longer period. While employment status showed univariate association with EMA use during the follow-up, its effect disappeared in multivariate models. Furthermore, exploratory analyses suggested that retention increased with age over time, although it slightly decreased in individuals aged 70 years and older compared with those aged 60–70 years.

Of the 512 patients who agreed to participate, 387 (75%) installed the EMA application at baseline. This rate is slightly lower than that reported by Porras-Segovia et al. (2022) in the SmartCrisis 1.0 study (81.2%) [21], but similar to levels found with passive EMA [12]. A feasibility study conducted during the COVID-19 outbreak found that 67% of older adults (mean age 65 years) installed and used an active EMA application over one week [36]. In younger individuals, adherence to EMA—defined as daily survey completion—reached 90% in the first week [37]. Other studies have also examined EMA feasibility across psychiatric populations, although most assessed compliance as the proportion of answered EMA prompts relative to the total delivered, with rates ranging from 52% to 90% [14].

In our study, we decided to assess the installation rates of the application at baseline as the primary outcome. In fact, intalling the application constitutes a critical prerequisite and a potential barrier to patients’ access to the active EMA. Furthermore, it constitutes the first step in enrolling vulnerable population at risk of suicide into a close monitoring procedure, potentially enabling improved health outcomes.

While some people may not enter the mental healthcare system for fear of stigma, the digital divide may also exclude individuals with psychiatric vulnerabilities [38]. Therefore, understanding the characteristics associated with access to EMA technologies is essential to reach patients with the highest preventive needs. We characterized the specific profile of patients who installed the EMA application: they had a higher level of education, were more likely to be unemployed, and more likely to be diagnosed with depression compared with those not using EMA. It is possible that unemployed participants may include people with mental-related disability, which may explain the coexistence of this factor with depressive disorders in the patient’s profile. This should be explored in future studies. Importantly, these features are shared with populations at increased risk of suicide [39]. Indeed, meta-analyses have reported increased odds for suicidality in unemployed people [40], particularly in those with a history of mental disorders [41]. Furthermore, depressive disorders are associated with suicidal ideation and death by suicide [42]. In the same line, higher education attainment may increase the risk of death by suicide [43]; [44]. Patients with such profile may be more likely to accept an offer of additional mental health resources.

To achieve their preventive goals, EMA monitoring programs must retain the most vulnerable individuals during the 3 months following a suicide attempt. In fact, suicide risk is highest during this period [31], particularly among depressed people following hospital discharge [32]. Individuals aged ≥50 years and those in an intimate relationship were most likely to use EMA for more than 90 days.

The risk of death by suicide increases with age [1], and people aged 50 years and older may be less likely to benefit from suicide prevention interventions, including safety planning, referral to mental health services, and psychiatric hospitalization after a suicide attempt [10]. Hence, longer retention of patients aged ≥50 years in EMA monitoring may help compensate for barriers to healthcare access. Although the age-related digital divide is thought to prevent older people from using digital tools for healthcare, we observed increasing retention rates with age. In fact, recent studies reported increasing smartphone use by older people [45] and its internet-related services. While older people may refrain from smartphone use due to feeling of incompetence or negative attitudes toward new technologies, Vassilakopoulou et al. (2023) outlined that emotional and cognitive support from family or partner may encourage learning and willingness to adopt digital tools [15]. Furthermore, a recent meta-analysis outlined elevated compliance rates to EMA programmes in the individuals aged 60 an older [18]. This is in line with our results as we have shown that older age, along with being in an intimate relationship, are both likely to increase retention in EMA. We did not find significant interactions between both variables probably due to lack of power.

Although the 50-year age cutoff may be debated, as it was set a priori based on the potential effects of the digital divide and generational differences in smartphone use, the trend toward increased retention with age was confirmed by exploratory analyses. Retention in EMA increased across the lifespan, with the highest rates observed in patients aged 60 years and older—approximately twice those of patients under 35 years. However, retention slightly declined among individuals aged 70 years and older compared with those aged 60–70 years. Nevertheless, the retention rates in this group remaind higher than in those aged between 50 and 60 years. This observation could encourage clinicians to propose EMA to the elderly as they have often poor access to psychiatric facilities [22].

The use of active EMA opens the way for tailored suicide prevention through ecological momentary interventions involving the implementation of digital therapeutic tools [24]. Future studies should include populations with rare access to the internet or to smartphones to investigate the specific barriers impacting EMA participation. On a second level, EMA retention is crucial to ensure the efficacy of suicide preventive measures during high-risk periods. Therefore, interventions aiming at improving retention (such as personalized user support, streamlined app interfaces, or tailored motivational reminders) should be studied. Finally, other clinical populations may benefit from smartphone-based EMA. For instance, recent pilot studies indicate that active smartphone-based data collection may be feasible in forensic outpatients and may improve emotional awareness [46]. Further, Ross and colleagues emphasized the importance of contextual factors linked to digital inequalities in facilitating early engagement with mobile health programs implemented in forensic settings. [47].

### Limitations and strengths

This study assessed installation rates of the EMA application rather than compliance or response rates to EMA prompts, limiting conclusions about sustained engagement. Because participants did not receive financial incentives, installation may rather reflect intrinsic motivation and patient’s interest toward EMA. Although focusing on installation facilitates comparisons between active and passive EMA approaches [33]; [21], future studies should examine compliance to better understand factors influencing application use over time. Reasons for failed installation may vary (e.g., incompatible device, refusal, technical problems) and should have been documented. Differences in digital literacy may also influence EMA use, as could cultural and socioeconomic factors such as income or place of residence (urban vs rural). Education level and employment status were highly collinear, so their associations with application installation at baseline were tested in separate logistic regression models to improve interpretability. Some clinical variables had high levels of missing data. Missingness differed between EMA participants and non-participants only for employment status (80.8% vs 19.2%, p = 0.034) and impulsivity levels (65% vs 35%, p < 0.001), but not between those who used the application for more than 90 days and those who used it for less than 90 days.

Strengths of this study include the large sample of well-characterized individuals from two EMA cohorts (SmartCrisis 1.0 and 2.0) following suicide attempts or ideation. To our knowledge, both the sample size and the 90-day retention period are among the largest reported in EMA research.

## Conclusions

In this study, age and clinical characteristics influenced participation and retention in active EMA among patients at risk of suicide. Higher education, unemployment, and major depressive disorder were associated with greater participation, whereas older age and being in an intimate relationship predicted longer retention. Individuals aged ≥50 years were most likely to maintain EMA use beyond 90 days, indicating that older adults can effectively participate in digital monitoring despite potential barriers. These findings support the feasibility of smartphone-based EMA as a complementary approach to suicide prevention, particularly in older adults, by enabling real-time monitoring and early intervention. Future research should address barriers to application use (such as digital literacy, cultural factors or cognitive impairment) and optimize digital interventions for populations with the greatest prevention needs.
